# Rheumatoid arthritis‐associated interstitial lung disease hotspots and future directions: A Web‐of‐Science based scientometric and visualization study

**DOI:** 10.1002/iid3.944

**Published:** 2023-08-28

**Authors:** Yue Yang, Zixuan Zhang, Xieyu Zhang, Xinwen Zhang, Kai Zhi, Xin Zhao, Jiahe Zhao, Wei Cao

**Affiliations:** ^1^ Wangjing Hospital of China Academy of Chinese Medical Sciences Beijing China; ^2^ Department of Rheumatology Guang'anmen Hospital Beijing China; ^3^ China Academy of Chinese Medical Sciences Beijing China

**Keywords:** iInterstitial lung disease, pathogenesis, predictive and prognostic factors, rheumatoid arthritis, treatment, visualization

## Abstract

**Objective:**

To identify new trends and potential hotspots in research on rheumatoid arthritis‐associated interstitial lung disease (RA‐ILD).

**Materials and Methods:**

The Web of Science (WOS) database was used to search for RA‐ILD‐related literature published between August 31, 2002 and August 31, 2022. CiteSpace 6.1.R3, VOSviewer version 1.6.17, Scimago Graphica, and Pajek V2.0 visualization software were used to conduct a comprehensive analysis and network visualization mapping of the authors, countries, institutions, journals, cited references, and keywords.

**Results:**

A total of 2412 articles were retrieved, and the number of articles published has grown annually since 2002. Eric L. Matteson was the most prolific author, and the Mayo Clinic and UNITED STATES have the highest publishing volume and influence. *Clinical Rheumatology* is the journal with the most papers published. *Rheumatology* was the most cited journal. The citation clusters and keywords concentrated on the mechanism, treatment, and predictive and prognostic factors.

**Conclusion:**

Pathogenesis, treatment, and predictive and prognostic factors were among the RA‐ILD research directions and hotspots. Antirheumatoid drugs, especially biologics and small molecule inhibitors, were among the most actively researched treatment options. The results of this study provides an in‐depth understanding of the development of RA‐ILD publications, aids researchers in understanding hotspots and trends and provides a new perspective for future RA‐ILD research.

## 1 INTRODUCTION

Rheumatoid arthritis (RA) is a systemic autoimmune disease. Although arthritis is the most frequent RA symptom, extra‐articular symptoms may occur in 50% of the RA population.^1^ Interstitial lung disease (ILD) is considered to be the most common extra‐articular manifestation. It worsens the disease prognosis and is detected in up to 60% of patients with RA, with clinically significant illness occurring in 10% of cases.^2^, ^3^ Early diagnosis of RA‐ILD remains difficult despite the increased awareness of RA‐ILD. It continues to be a leading cause of mortality with a median survival of only 3–7 years after diagnosis.^3^, ^4^ The median survival was 2.6 years for patients with the usual interstitial pneumonia (UIP) pattern, the most prevalent histological type of RA‐ILD.^3^

Researchers have begun to pay more attention to RA‐ILD in view of the above‐mentioned factors. However, some controversies remain regarding RA‐ILD, including pathogenesis, treatment, predictive markers, and prognostic factors.^5^ RA‐ILD limits the therapeutic strategy especially with regard to the medication. In addition, biological agents also bring new challenges to RA‐ILD. Numerous research studies on RA‐ILD have been published related to these issues. It is becoming increasingly challenging for researchers, particularly novice investigators, to properly comprehend, assess, and pinpoint the most pertinent and valuable information in the field due to the rapid growth of publications. Therefore, a macro description of research hotspots, trends, high‐impact publications, organizations, and authors in this field is required to help new researchers. Scientometrics analysis is an increasingly popular method to obtain the above‐mentioned parameters. This method allows for a quantitative and qualitative assessment of previous scientific accomplishments and the current state of a particular area of study. Neurology, cancer, and cardiovascular medicine are just few of the medical specialties that make frequent use of this technique.^6^, ^7^, ^8^ As regards to RA, there has been visualization of the knowledge structure related to RA‐related cardiovascular disease and osteoporosis.^9^, ^10^ There is a gap in our understanding of the leading cause of death among people with RA, and more research is needed in the field of RA‐ILD. In this study, we analyzed all of the research papers published on RA‐ILD from August 31, 2002 to August 31, 2022 to determine the current state of the field, map out the existing body of knowledge, and project its potential for future growth.

## 2 MATERIALS AND METHODS

### 2.1 Data sources and search strategies

Data were retrieved and collected from the Web of Science Core Collection (WOSCC) database. The search strategy was as follows: (((TS = (rheumatoid arthritis) or TS = (rheumatic arthritis) or TS = (rheumatism arthritis) or TS = (rheumatoid arthritis)) and (TS = (interstitial lung* disease*) or TS = (interstitial pneumonia*) or TS = (“ILD”) or TS = (pulmonary fibros*) or TS = (pulmonary fibrot*) or TS = (lung fibros*) or TS = (lung fibrot*) or TS = (alveoli* fibros*) or TS = (alveoli* fibrot*) or TS = (pulmonary sarcoid*) or TS = (pulmonary granuloma*) or TS = (lung sarcoid*) or TS = (lung granuloma*))) and LA = (English)) and DT = (Article or Review). The language was English, the literature type was article and review, and the publication date was set from August 31, 2002 to August 31, 2022.

### 2.2 Data extraction and analysis

Visualization software CiteSpace 6.1.R3, VOSviewer version 1.6.17, Scimago Graphica, and Pajek V2.0 were used for scientometrics analysis. The following information was extracted through the above software: authors, countries, institutions, journals, references, and keywords. Two researchers conducted data extraction to guarantee the reliability and correctness of the research findings. If the two researchers had different findings, they discussed the issue until a conclusion was reached.

**Figure 1:**
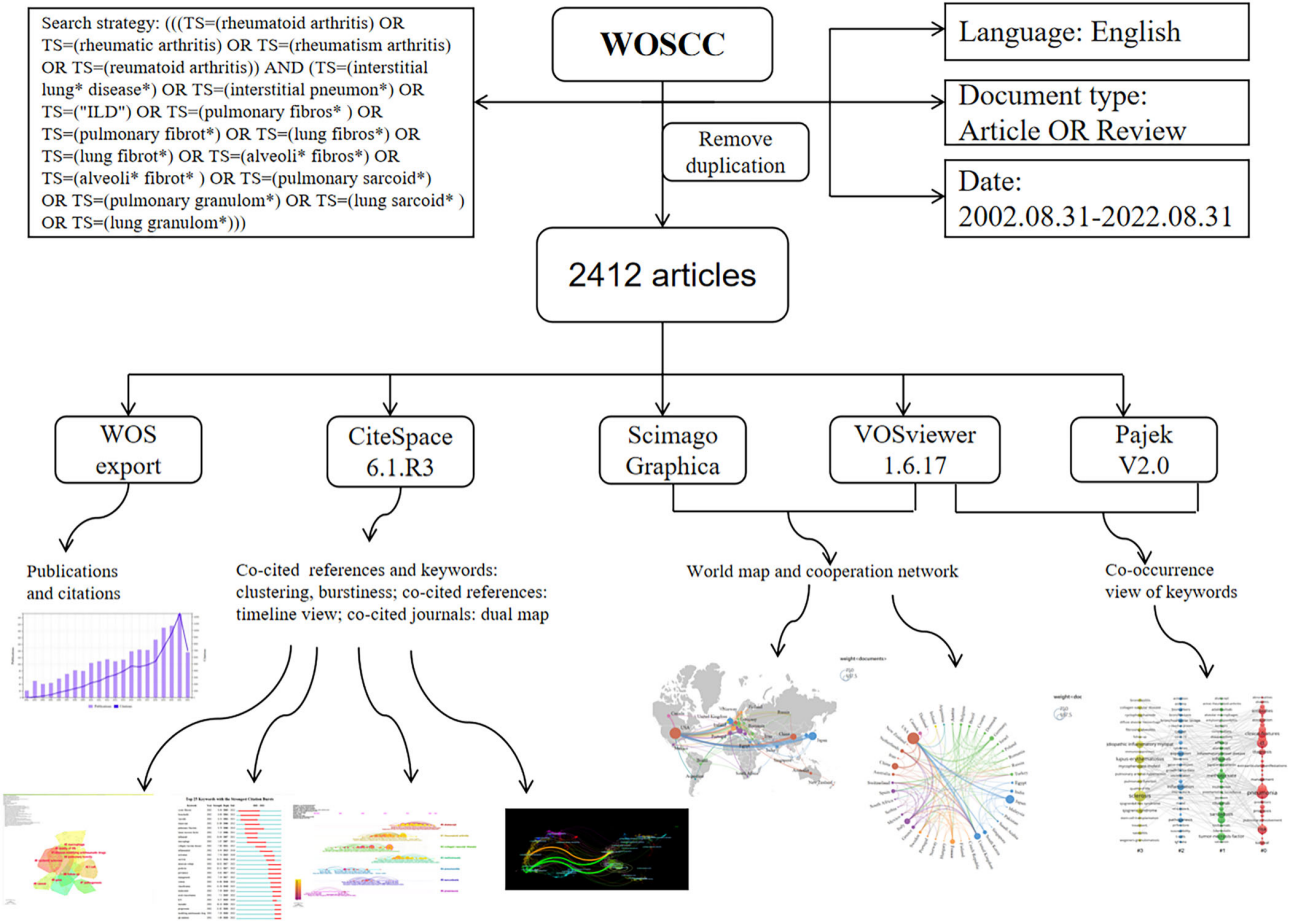
Strategy for selecting and mapping literature.

The use of CiteSpace included: clustering, timeline view, and burst detection analysis of co‐cited references, journal dual map analysis, clustering and burst analysis of keyword detection. VOSviewer was employed for a scientometrics study of authors, nations, and institutions. Specifically, VOSviewer was combined with the Scimago Graphica and Pajek programs to map the global distribution of national contributions and cross‐country cooperation networks, and to show the co‐occurrence analysis of keywords. Figure 1 displays the unique literature selection process and mapping.

## 3 RESULTS

### 3.1 Publication and citation trends

**Figure 2:**
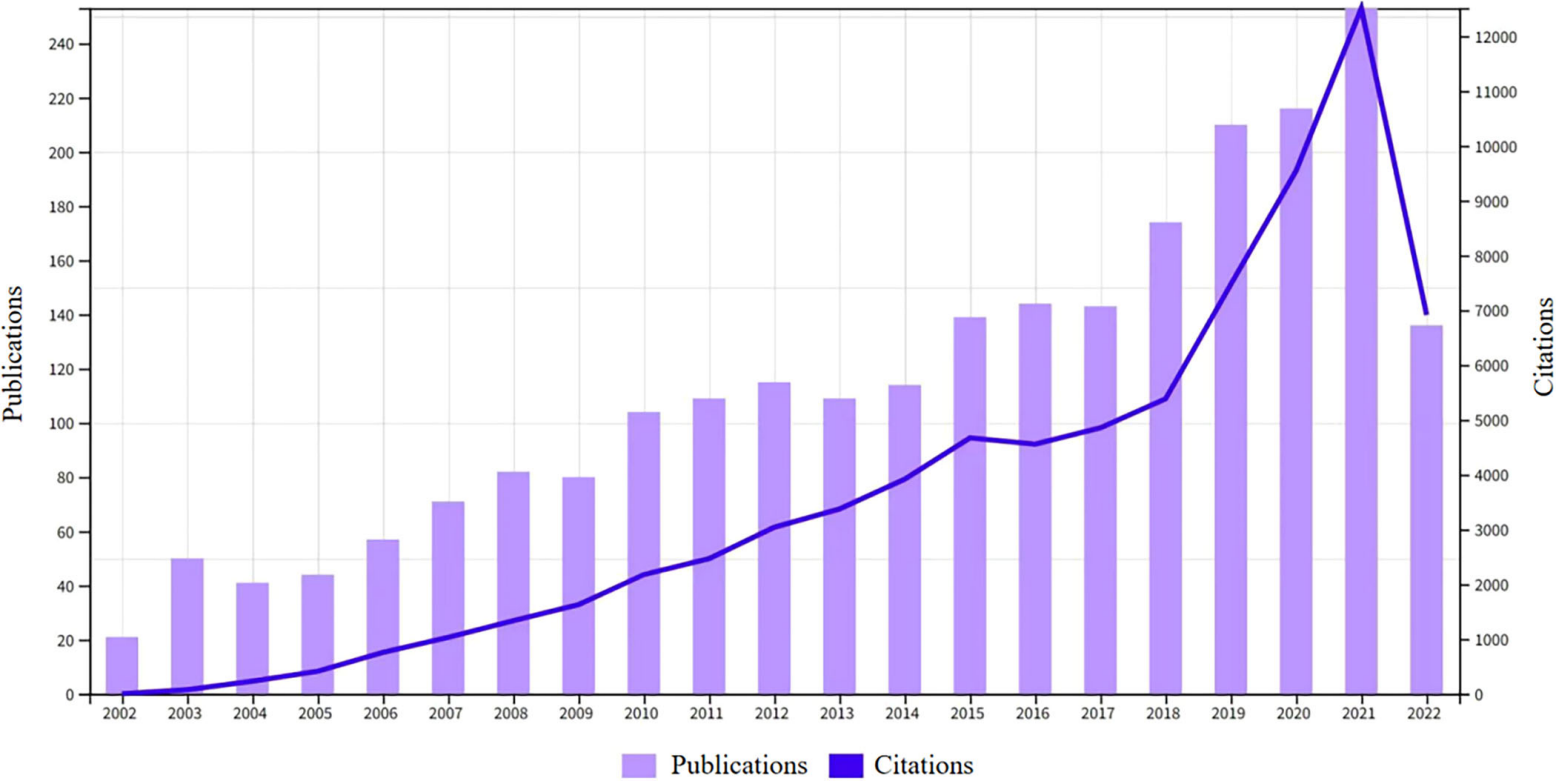
Trends in the growth of publications and citations from August 31, 2002 to August 31, 2022.

We can assess the patterns and rate of research in this field based on the number of publications in each period. The retrieval approach revealed that WOSCC had collected 2412 publications on RA‐ILD (no duplicates were found), with a total of 63,428 citations (self‐citations removed). Each article received an average of 31.12 citations and the H‐index score was 120, which is a crucial measure of a researcher's scientific impact. Since 2022 was not an entire year, the rise only covers the years before 2021, displayed in Figure 2. The annual number of publications and citations also showed an increasing trend annually, and peaked in 2021. This indicates that the research field has grown in popularity and continues to grab the interest of the academics.

### 3.2 Analysis of authors

**Figure 3:**
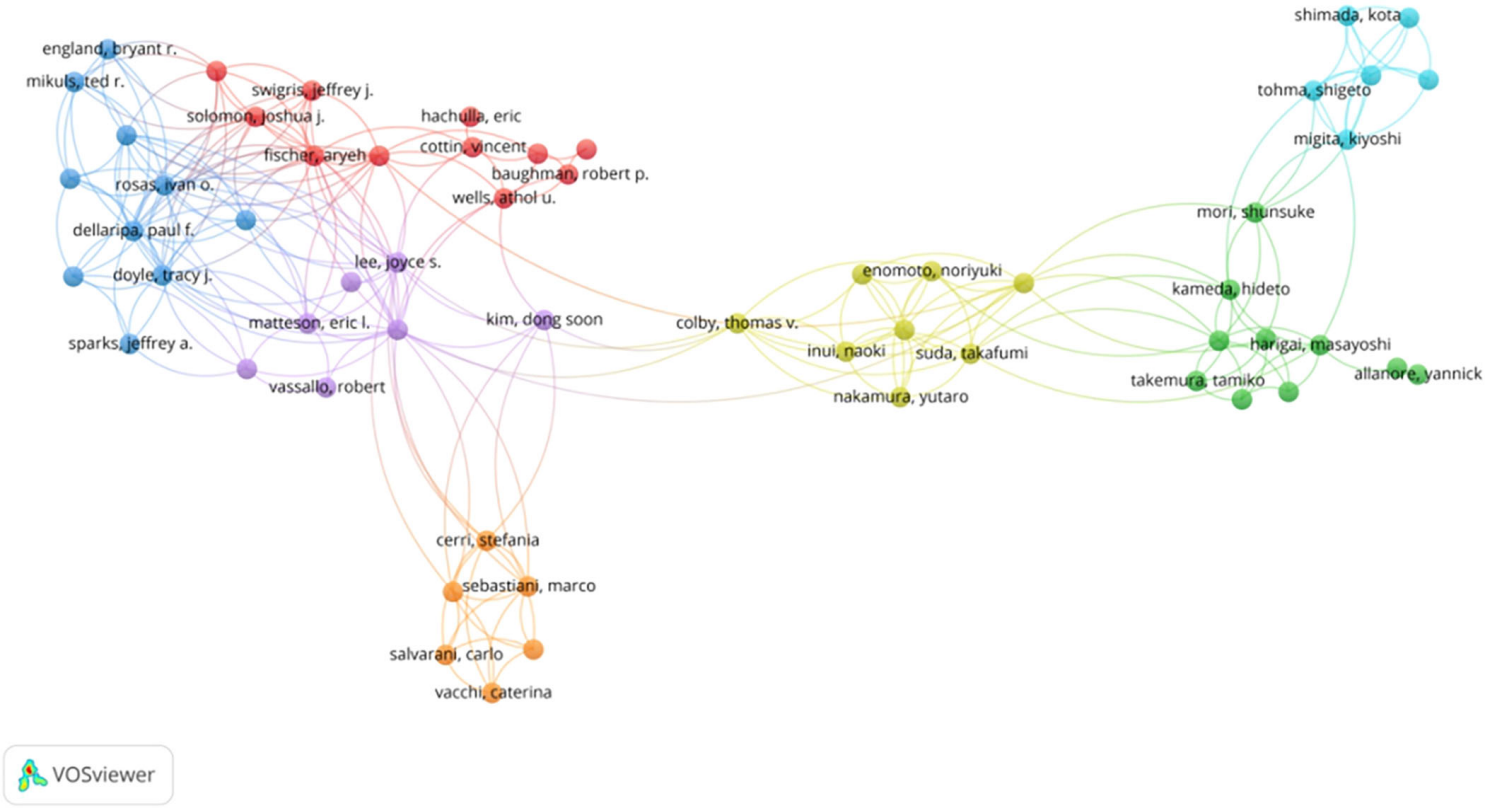
Collaboration networks between researchers.

The co‐authorship analysis of authors is displayed using the VOSviewer program (Figure 3). Table 1 lists the top 10 most prolific authors. Among them Eric L. Matteson (28 articles) from the Mayo Clinic College of Medicine and Science ranked first. We can see from the figure that there are seven major collaborative groups, and it is noteworthy that three of the researcher groups are from the United States. The author with the highest overall linkage intensity was Takafumi Suda (78) from Hamamatsu University School of Medicine, Japan, which indicated that this researcher played an active bridging role in institutional collaborations.

**Table 1:** The citations and total link strength of top 10 authors, countries, and institutions.

Rank	Authors	Citations	Total link strength	Countries	Citations	Total link strength	Institutions	Citations	Total link strength
1	Eric L. Matteson	28	39	USA	750	454	Mayo Clin	96	78
2	Paul F. Dellaripa	27	77	Japan	396	109	Natl Jewish Hlth	56	65
3	Tracy J. Doyle	26	71	United Kingdom	236	296	Brigham & Womens Hosp	47	59
4	Aryeh Fischer	26	38	China	232	93	Univ Colorado	43	51
5	Takafumi Suda	21	78	Italy	189	243	Univ Cincinnati	39	14
6	Ivan O. Rosas	21	67	France	137	220	Harvard Med Sch	37	33
7	Jay H. Ryu	21	48	Germany	111	224	Royal Brompton Hosp	35	31
8	Kevin K. Brown	20	47	Spain	92	144	Johns Hopkins Univ	34	26
9	Robert P. Baughman	20	9	South Korea	92	59	Univ Michigan	32	45
10	Athol U. Wells	20	8	Netherlands	80	189	Univ Manchester	31	46

### 3.3 Analysis of countries

**Figure 4:**
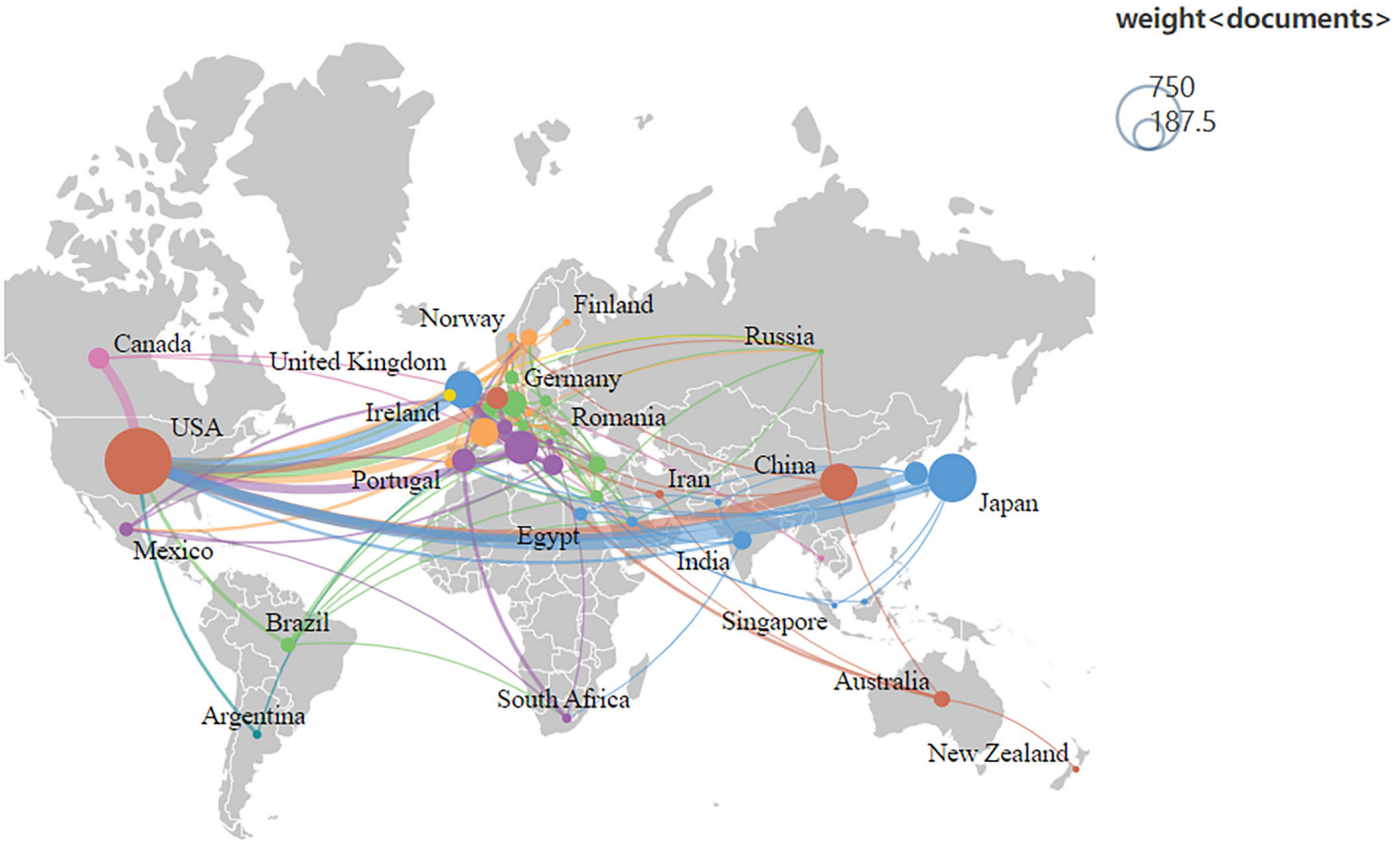
World map of each country's contribution.

**Figure 5:**
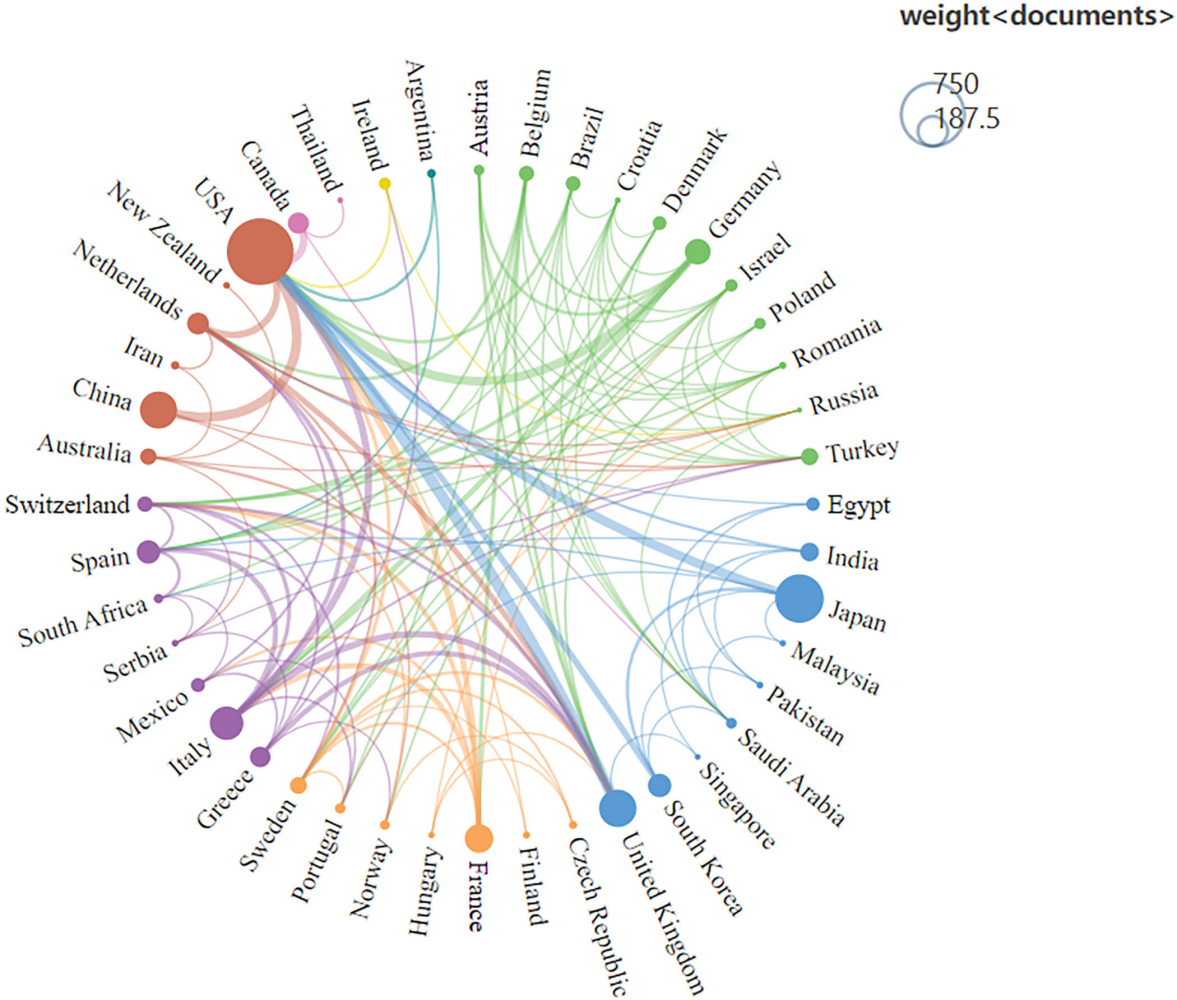
International cooperation network.

The map of the countries that have contributed to RA‐ILD research is depicted in Figure 4. The chart shows that authors in North America, Europe, and East Asia are responsible for most publications. Specifically, the United States has the most publications in this field, as seen in Table 1. The intercountry cooperation network depicts the international collaboration between many nations (Figure 5). The degree of intercountry collaboration is shown by the thickness of the lines connecting the two countries. Based on the overall strength of the connecting lines it is evident that the United States (454) has a dominant influence in this field and collaborates with many countries. Among them, the United States cooperates most closely with the United Kingdom, Japan, China, and Germany.

### 3.4 Analysis of institutions

**Figure 6:**
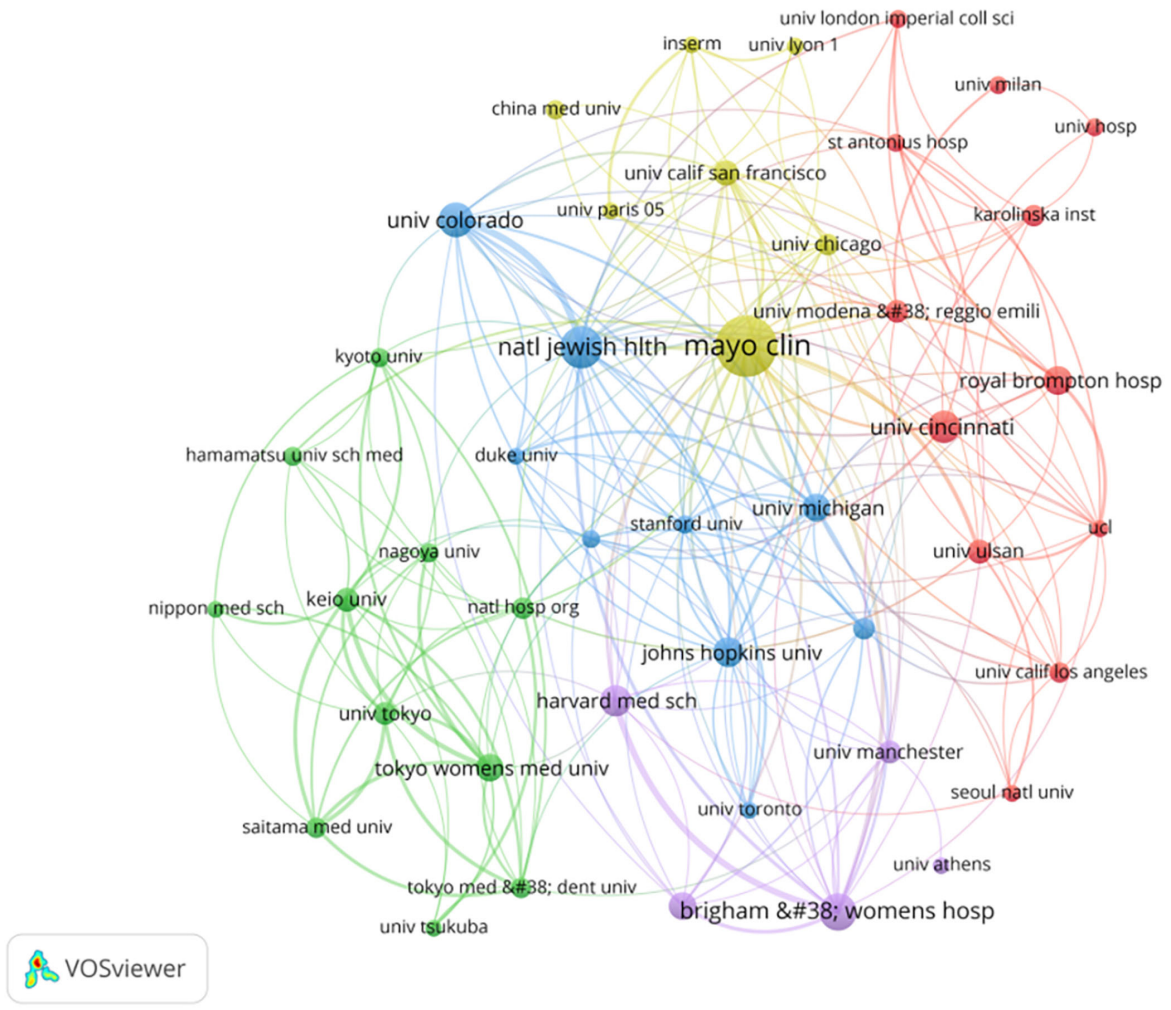
Interinstitutional cooperation networks.

Based on the literature findings, 590 institutions contributed to this field over the past 20 years. The top 10 most productive institutions, all from the United States, are shown in Figure 6. When the institutions in Table 1 are combined, Mayo Clinic (96 papers) appear on top with respect to publications and total linking intensity (78). Interinstitutional collaboration may be found to be dispersed throughout high‐income nations like North America and Europe.

### 3.5 Analysis of journals and co‐cited journals

In this study, 2412 articles were analyzed, which were published in 720 different journals. The top 10 active journals published 470 articles on RA‐ILD (Table 2), accounting for 19.50% of all articles. The most published articles were on clinical rheumatology (72 articles [2.99%], IF = 3.650). The impact of journals depends on the number of times they are co‐cited. Twelve journals have been cited more than 1000 times. Specifically, the journal with the most citations was *Rheumatology* (3250).

**Table 2:** The top 10 journals ranked by number of citations.

Rank	Journals	Number of documents	Total citations	Percent (%)	Impact factor (2022)	2022 JCR partition
1	*Clinical Rheumatology*	72	1369	2.99	3.650	Q3
2	*Rheumatology*	67	3250	2.78	7.046	Q1
3	*Modern Rheumatology*	56	683	2.32	2.862	Q4
4	*Respiratory Medicine*	45	1745	1.87	4.582	Q2
5	*Journal of Rheumatology*	42	1753	1.74	5.346	Q2
6	*Seminars in Respiratory and Critical Care Medicine*	42	883	1.74	3.921	Q2
7	*Rheumatology International*	39	743	1.62	3.580	Q3
8	*Clinical and Experimental Rheumatology*	38	726	1.58	4.862	Q2
9	*Plos One*	35	916	1.45	3.752	Q2
10	*Arthritis Research & Therapy*	34	1710	1.41	5.606	Q1

**Figure 7:**
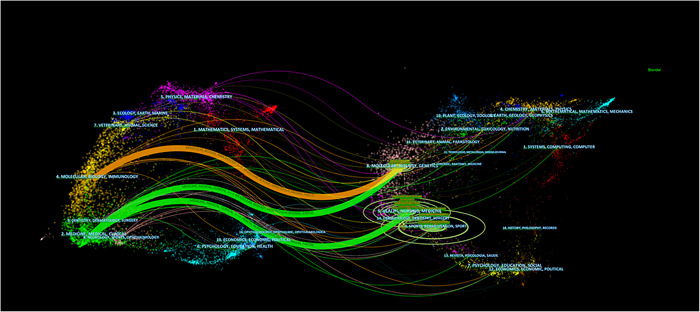
The dual‐map overlay of citing of article citation relationship.

In addition, we constructed the journals dual map overlay of RA‐ILD based on Citespace software (Figure 7). The journal overlay map and the topical distribution of academic publications is shown as a dual map. On the left is a map of journals with citations, and on the right is a map of journals that have been cited. Generally, achievements in this field are concentrated in journals related to biology, molecular science, immunology, and so on. However, the most cited articles were published in journals in the fields of nursing, molecular, biology, and so on. The three thickest lines in the map identify the main reference paths. Yellow and green paths indicate that research published in molecular/biology/genetics or health/nursing/medical journals were usually cited in molecular/biology/immunology or medical/medical/clinical journals.

### 3.6 Analysis of co‐cited references

The literature with a high co‐citation frequency is often regarded as the most valuable and influential research in a particular field. The top 10 references on RA‐ILD that have been referenced the most are shown in Table 3. Most of the co‐cited references in the top 10 were published in rheumatology journals.

**Table 3:** Top 10 co‐cited references.

Author	Year	Article title	Journal	Total citations
Kelly Clive A, et al.	2014	Rheumatoid arthritis‐related interstitial lung disease: associations, prognostic factors and physiological and radiological characteristics‐‐a large multicentre UK study.	*Rheumatology*	103
Joshua J. Solomon, et al.	2016	Predictors of mortality in rheumatoid arthritis‐associated interstitial lung disease.	*Eur Respir J*	103
Charlotte Hyldgaard, et al.	2017	A population‐based cohort study of rheumatoid arthritis‐associated interstitial lung disease: comorbidity and mortality.	*Ann Rheum Dis*	92
P.‐A. Juge, et al.	2018	MUC5B Promoter Variant and Rheumatoid Arthritis with Interstitial Lung Disease.	*New Engl J Med*	80
Md Yuzaiful Md Yusof1, et al.	2017	Effect of rituximab on the progression of rheumatoid arthritis‐related interstitial lung disease: 10 years' experience at a single centre.	*Rheumatology*	76
K.R. Flaherty, et al.	2019	Nintedanib in Progressive Fibrosing Interstitial Lung Diseases.	*New Engl J Med*	76
Tim Bongartz, et al.	2010	Incidence and mortality of interstitial lung disease in rheumatoid arthritis: a population‐based study.	*Arthritis Rheum‐us*	70
Ganesh Raghu, et al.	2011	An official ATS/ERS/JRS/ALAT statement: idiopathic pulmonary fibrosis: evidence‐based guidelines for diagnosis and management.	*Am J Resp Crit Care*	67
Aryeh Fischer, et al.	2015	An official European Respiratory Society/American Thoracic Society research statement: interstitial pneumonia with autoimmune features.	*Eur Respir J*	67
Ganesh Raghu, et al.	2018	Diagnosis of Idiopathic Pulmonary Fibrosis. An Official ATS/ERS/JRS/ALAT Clinical Practice Guideline	*Am J Respir Crit Care Med*	63

**Figure 8:**
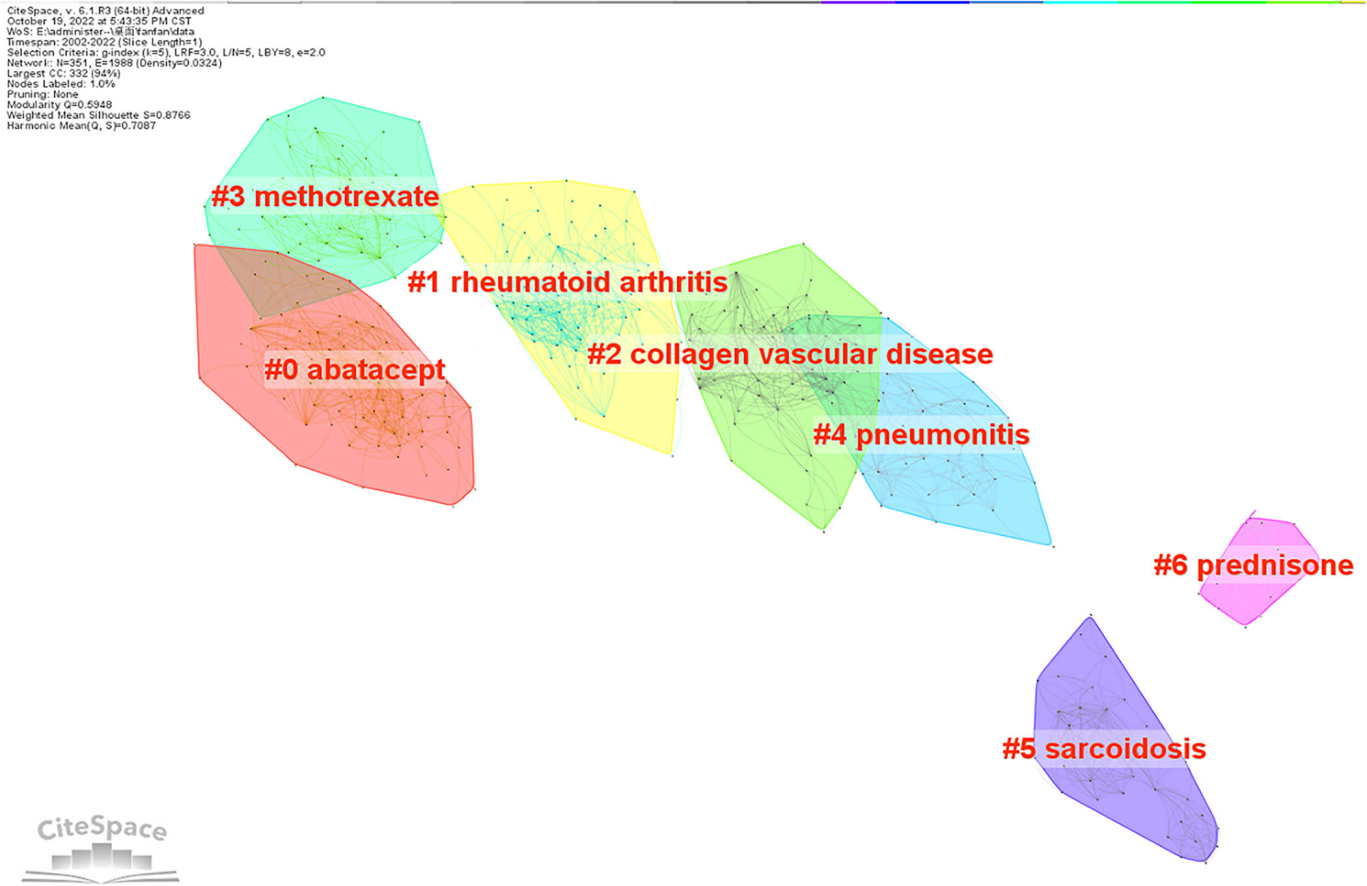
Clustering map of co‐cited references.

**Figure 9:**
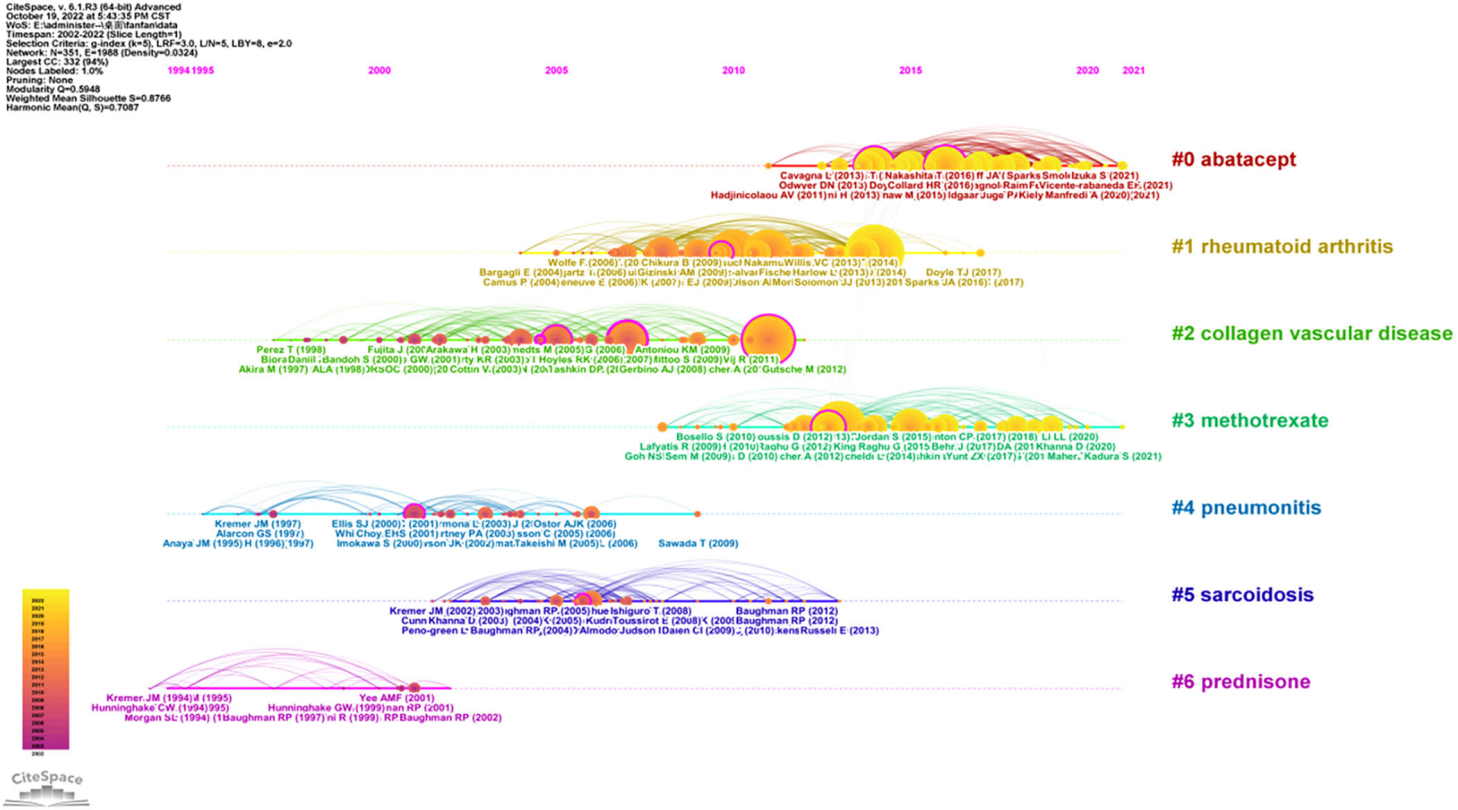
Timeline view of co‐cited references.

As shown in Figure 8, the co‐cited references were divided into seven different clusters. Abatacept (#0) was the largest class, followed by rheumatoid arthritis (#1), collagenous vascular disease (#2), methotrexate (#3), pneumonia (#4), nodular disease (#5), and prednisone (#6). Based on this, we plotted the corresponding timeline view of the references (Figure 9) to further understand the evolutionary characteristics of each cluster. We can observe from the figure that the research focus of RA‐ILD has shifted sequentially from disease characteristics (#1, #2, #4, #5) to antirheumatoid drug therapy (#0, #3, #6), indicating that antirheumatoid drug research in RA‐ILD has been a hotspot from the last 20 years.

### 3.7 Analysis of keywords

**Figure 10:**
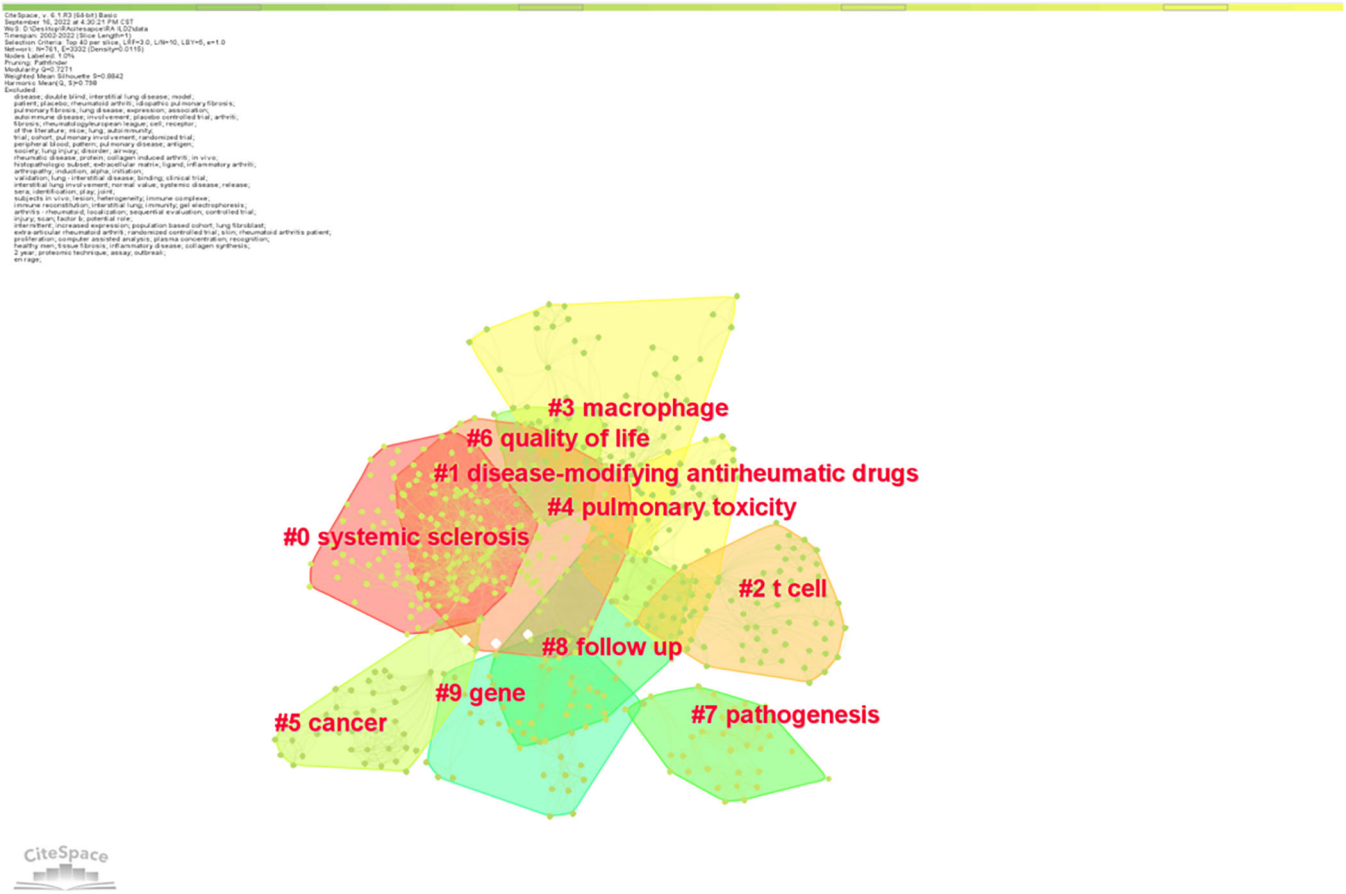
Keyword clustering map.

We removed the words with no significance and merged the terms with the same meaning (see Supporting Information: 1). The clustering analysis of the network knowledge map of keywords yielded 10 valid clusters, as shown in Figure 10. It can be seen that there were several clusters overlapping in the keywords clustering network, indicating that some of the clusters were closely related, although there were differences among the studies and the research topics were more concentrated. The focus of RA‐ILD research can be divided into three categories: mechanism exploration (#0, #2, #3, #7, #9), pharmacotherapy (#1, #4), and prognosis (#5, #6, #8).

**Figure 11:**
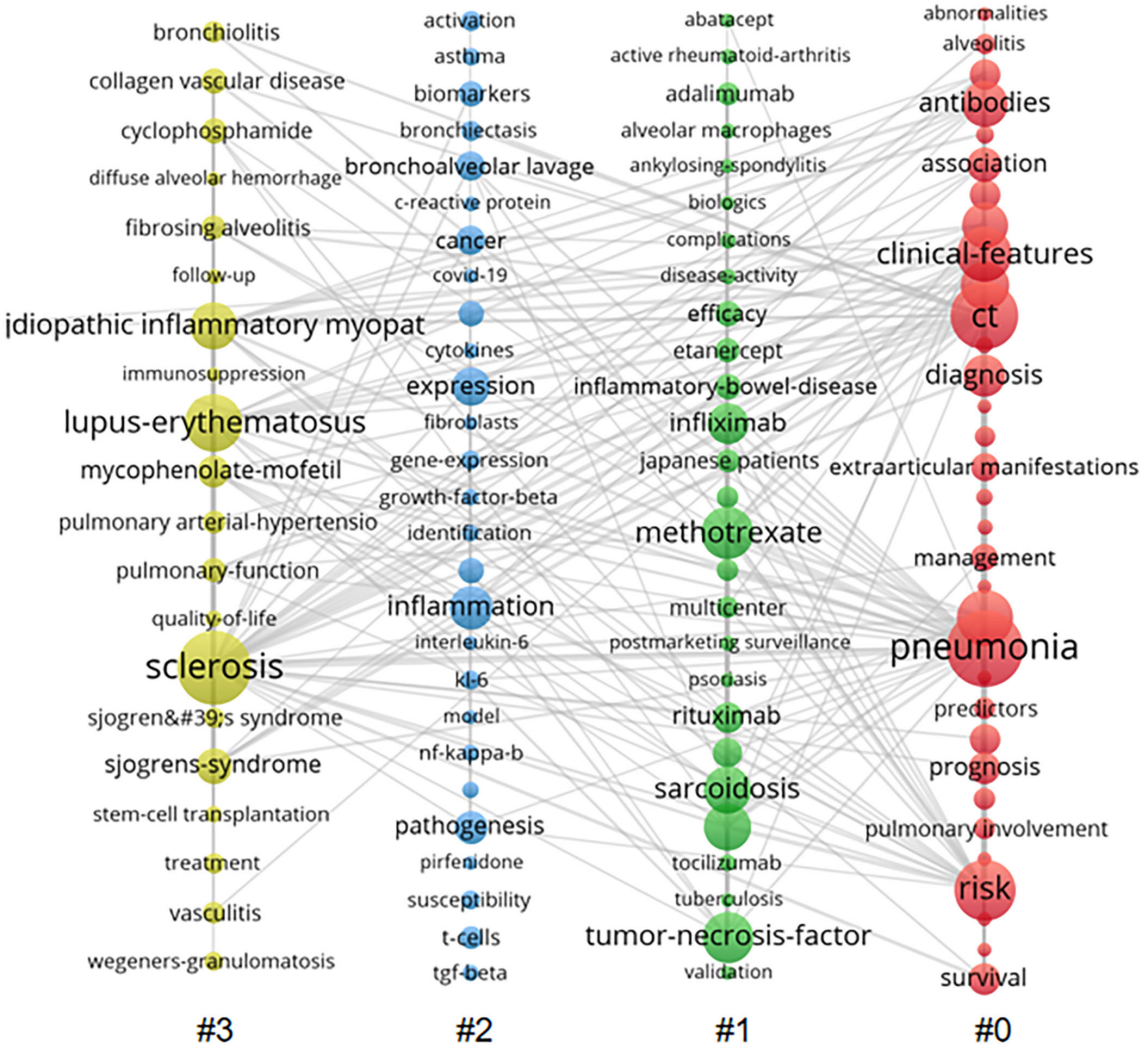
Keyword co‐occurrence view.

The co‐occurrence visualization of the terms that appeared more than 21 times concurrently in RA‐ILD research is shown in Figure 11. Each column is a cluster that VOSviewer created; the frequency of the keyword determined the size of the node, and the thickness of the connection showed how often two nodes occur together; the more profound the linkage, the more frequently the co‐occurrence occurred.

**Figure 12:**
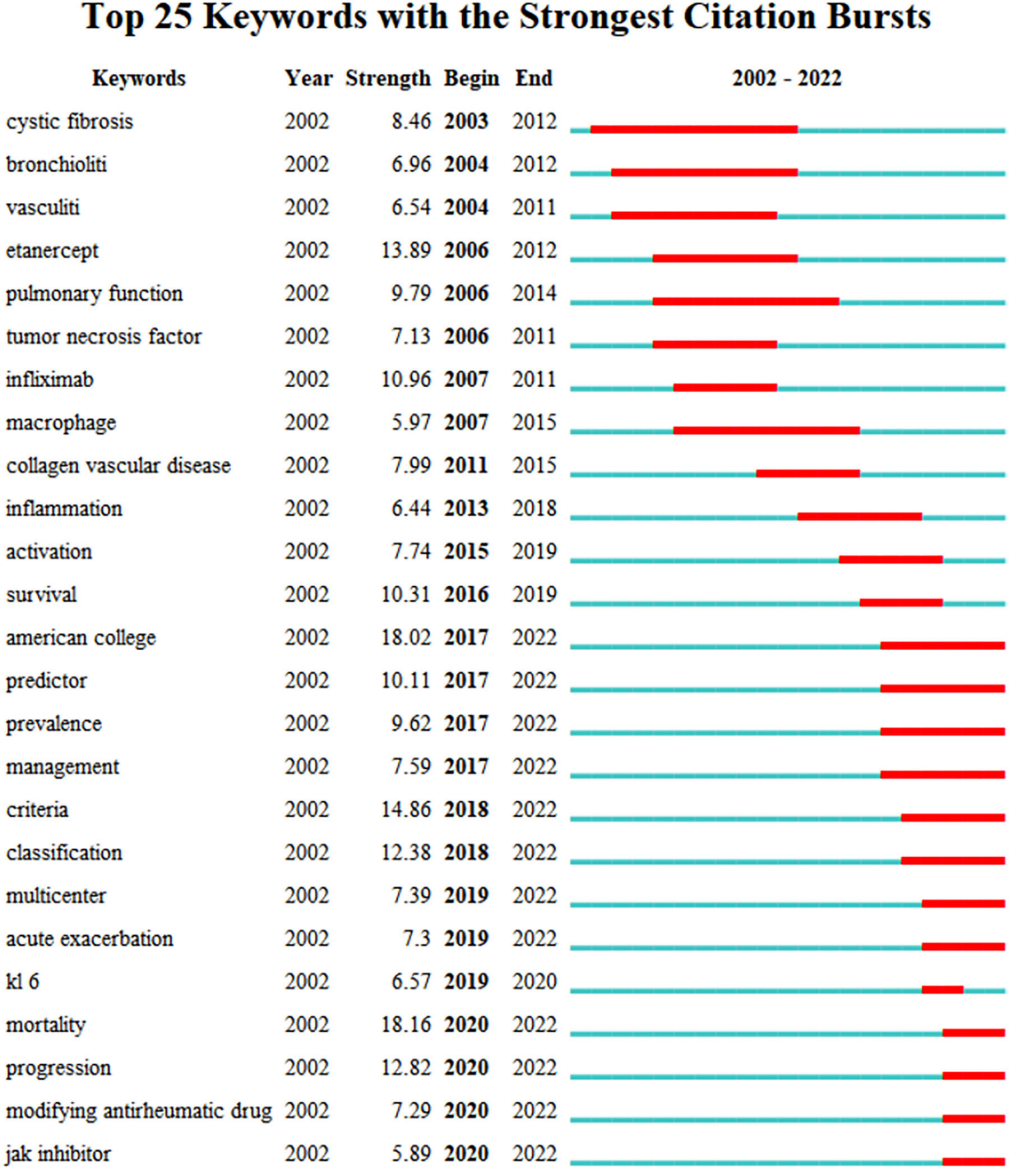
Keyword burstiness analysis.

Keyword burstiness refers to a considerable increase in the frequency of keywords within a short period, and was used to quickly understand the research being done at this time and to pinpoint where new research was being conducted; Figure 12 shows the top 25 terms. The findings revealed that the most fantastic burst keyword during the previous 2 years was jak inhibitor.

## 4 DISCUSSION

In this study, we conducted a scientometrics analysis of RA‐ILD research from August 31, 2002 to August 31, 2022. We discovered that RA‐ILD saw a growing number of publications and citations throughout the previous two decades (Figure 2). In light of this, we believe that RA‐ILD has garnered much attention from academics and is a significant research area globally.

We extracted 2412 articles on RA‐ILD from WOSCC using the literature screening approach outlined previously. The development of RA‐ILD has benefited greatly from the cooperation between countries. Six European countries, 1 American country, and 3 Asian countries are among the top 10 nations. Research is dominated by the United States, Japan, and the United Kingdom, with the United States topping the list for publication volume and total link strength. Most of the top 10 institutions are in the United States, indicating that the United States is a key player in RA‐ILD research with close international collaboration. The United States' leadership is a result of the contributions from research organizations like the Mayo Clinic and National Jewish Health. China is the only developing nation in the top 10 nations in terms of the number of publications, indicating that China has made significant advancements in the field of RA‐ILD research over the past 20 years. Notably, the majority of excellent research originates from industrialized nations. In terms of health care and scientific research, low‐income and middle‐income nations lag developed nations, which calls for increased international cooperation.

According to the results of co‐cited references and keywords analysis, we further found that current research mainly focus on the following three aspects: pathogenesis of RA‐ILD, treatment of RA‐ILD, and prediction and prognostic factors of RA‐ILD.

### 4.1 Pathogenesis of RA‐ILD

The pathogenesis of RA‐ILD is not yet completely understood based on the existing studies. The co‐cited references provide evidence of the pathogenesis‐related commonalities between RA‐ILD and idiopathic pulmonary fibrosis (IPF). The main results showed that the risk factors may be related to age, smoking, anticitrullinated protein antibodies (ACPA), genetic variation, and so forth. Age is a separate risk factor for developing ILD in RA cohorts, with most RA‐ILD diagnoses occurring in the sixth decade of life.^11^ For people who have smoked for >25 years, the odds ratio for developing RA‐ILD was 3.8.^12^ Meanwhile, more studies have shown that ACPA and gene mutations are crucial for the onset and progression of RA‐ILD.

The citrullinated protein, also known as ACPA, generates auto‐antibodies. In studies, higher ACPA titers were associated with higher prevalence of ILD, even after adjusting for confounders, including RA and smoking.^13^ According to research, citrullination was discovered in the bronchoalveolar lavage fluid of RA‐ILD and IPF study participants. Approximately 50% of RA‐ILD patients have citrullinated proteins visible in their lung tissue.^14^ Meanwhile, a recent meta‐analysis revealed that the risk of RA‐ILD was significantly linked with the serum ACPA titer and the risk increased in ACPA‐positive patients when compared with ACPA‐negative individuals. In a region‐based subgroup analysis, the risk of RA‐ILD was significantly correlated with ACPA titers in Asian, European, and African populations but not in Americans.^15^ Therefore, ACPA is essential for the incidence and progression of ILD in RA patients.

On the genetic front, the major finding was the MUC5B mutation. A substantial genetic risk factor for the onset of IPF has been identified as the MUC5B promoter variation.^16^ Studies in the genetics of RA‐ILD have discovered genetic risk factors shared by RA‐ILD and IPF, which provided additional evidence that these two diseases may be related. The MUC5B promoter variant rs35705950 was associated with RA‐ILD development in a study of the Western population. However, East Asians, including Japanese and Chinese, rarely have this gene polymorphism. The MUC5B gain of functional single‐nucleotide polymorphism in rs35705950 was subsequently discovered to be substantially related to RA‐ILD.^17^ Additionally, recent research found that the incidence of ILD in patients with RA was 6.1% for MUC5B noncarriers and 16.8% for MUC5B carriers. At the age of 65 years, the difference in risks became apparent, with men having a larger risk.^18^

There was also an elevated chance of developing RA‐ILD due to other variables, such as certain human leukocyte antigen (HLA) alleles and blood biomarkers. Several HLA variations may be brought on by RA‐ILD, including HLA‐B54, HLA‐DQB1*0601, HLA‐B40, and HLA‐DR4.^19^ Up to 10% of sporadic IPF, 25% of familial IPF, and 10% of connective tissue disease (CTD)‐ILD were attributed to telomere‐related mutations.^20^ Bronchoalveolar lavage fluid from RA‐ILD patients had greater anti‐CCP antibody titers in paired samples when compared to serum.^21^ Additionally, patients with RA‐ILD had higher MMP‐7 levels than patients with RA without ILD. Furthermore, it has been demonstrated in cohorts of RA‐ILD in the United States that MMP‐7 value is inversely correlated with pulmonary function markers (forced vital capacity and carbon monoxide diffusing capacity, FVC and DLCO) and with worse dyspnea scores.^22^

### 4.2 Treatment of RA‐ILD

So far, therapy recommendations for RA‐ILD have mostly been based on trial data from IPF or other CTD‐linked ILDs, like systemic sclerosis‐associated ILD (SSc‐ILD). Meanwhile, the American College of Rheumatology (ACR) and the European League Against Rheumatism (EULAR) urged a multidisciplinary approach.^23^ When the therapeutic approach was chosen, antibacterial and anti‐inflammatory medications were most common in the acute/subacute form of RA‐ILD. In chronic RA‐ILD, RA‐associated arthritis must be quickly stabilized before the activity of ILD itself may be controlled, considering the safety of each antirheumatic medication used in RA‐ILD. Clinicians should consider initiating an antifibrotic medication if fibrosis is prevalent in patients with progressive RA‐ILD.^24^

The antirheumatic drug therapy for RA consists of both conventional and biologic disease‐modifying antirheumatic drugs (cDMARDs) (bDMARDs). However, drug‐related pulmonary illness, including lung infection, nodules, and ILD, has been associated with cDMARDs and bDMARDs.^25^ The top drug research listed in the keywords and references analysis findings are explored next.

In patients with RA, methotrexate (MTX) was associated with the development or exacerbation of ILD.^26^ However, some findings implied that MTX use was not associated to a higher incidence of RA‐ILD in patients.^27^ According to a meta‐analysis,^28^ MTX was associated with a higher risk of respiratory infections, but the risk of ILD was significantly lower than formerly reported. Kiely et al.^29^ discovered that MTX exposure was associated with a considerably lower incidence of RA‐ILD after conducting a multicenter prospective cohort study on 2701 patients with early RA. They also discovered that therapy prevented the onset of ILD in RA patients. Based on this evidence, we are confident that MTX may be helpful in the prevention and treatment of RA‐ILD. Leflunomide (LEF) use is contentious at the same time. According to a sizable observational study, 61 of 5054 patients with RA treated with LEF experienced new or worsening ILD.^30^ A systematic literature review revealed that LEF‐related ILD mostly occurred within the first 20 weeks after the initiation of therapy, and caused dyspnea in older patients, which could be fatal.^31^ However, according to a meta‐analysis, patients who received LEF treatment had a lower risk of noninfectious respiratory adverse events rather than an increased risk of respiratory adverse events.^32^ Patients with rapidly progressing fibrosis diseases may benefit the most from cyclophosphamide and mycophenolate mofetil (according to the results of the keywords analysis), both of which had positive effects on CTD‐ILD.^33^

Most conventional antitumor necrosis factor (TNF) medications have been documented to cause ILD, including infliximab, etanercept, adalimumab, golimumab, certolizumab pegol, and the IL‐6 receptor (IL‐6R) antagonist tocilizumab.^34^, ^35^, ^36^ However, most of the evidence on TNF inhibitor‐ILD is comprised of case reports. Additionally, research has demonstrated that abatacept was effective in the treatment of RA‐ILD.^37^ RA‐ILD was stabilized or improved in 88.6% of patients who were receiving treatment with abatacept.^38^ Rituximab (RTX) also demonstrated good clinical efficacy. A study examined the effectiveness of RTX treatment in 14 patients with RA‐ILD for more than a year; their forced vital capacity (FVC), forced expiratory volume in one second (FEV1), total lung capacity (TLC), and stable DLCO percentage all exhibited modest improvements.^39^ A similar finding indicated that the majority of pre‐RTX patients with ILD remained stable/improved after treatment during a significant follow‐up period.^40^ Studies using tyrosine kinase inhibitors, such as nintedanib and pirfenidone, have shown the value of antifibrosis therapeutic approaches. However, we need to monitor their adverse reactions during the treatment.^41^

Through the keywords burstiness, we can see that jak inhibitors have been the focus of attention at the forefront of treatment in recent years. According to in vitro research, JAK2 is a crucial intermediary molecule in TGF‐mediated myofibroblast trans‐differentiation, proliferation, and extracellular matrix protein synthesis. The regulating function of JAK2 in the etiology of pulmonary fibrosis was further demonstrated by inhibiting bleomycin‐induced mice pulmonary fibrosis with the JAK2‐selective pharmacological inhibitor CEP3377.^42^ Vacchi et al.^43^ described a patient with RA‐ILD who was successfully treated with tofacitinib. Retrospective studies found that jak inhibitors or abatacept treatment were associated with RA‐ILD stability or improvement in 83.9% and 88.6% of patients, respectively.^44^ According to a post‐hoc analysis of 21 clinical trials, the incidence rate of ILD events after tofacitinib treatment was 0.18.^45^ Furthermore, baricitinib treatment for RA patients is associated with a low risk of developing noninfectious ILD, similar to what has been observed with other jak inhibitors.^46^ It is anticipated that JAK inhibitors can boost hope for RA‐ILD patients as more research into them is conducted.

### 4.3 Predictive and prognostic factors of RA‐ILD

Those with RA‐ILD had a 2–10 times higher risk of mortality than those with non‐ILD RA, regardless of the length of the follow‐up period. It is evident that ILD is a serious complication for patients with RA, and its mortality rate is significantly higher than that of patients with RA without ILD.^47^ Therefore, it is very important to know the predictors and prognostic factors of RA‐ILD in advance for better diagnosis and treatment. RA‐ILD screening and monitoring are difficult in clinical practice. Majority of patients with RA‐ILD have no symptoms, and the best resources for early detection and ongoing follow‐up are limited. Furthermore, some patients may remain asymptomatic despite significant radiological abnormalities. If RA‐ILD is detected early, there may be a chance for early therapy and attentive follow‐up, which could stop the progression of ILD and enhance the long‐term result.^48^

Age, male sex, and history of smoking are all risk factors for RA‐ILD. Furthermore, high‐titer rheumatoid factors (RF) and ACPA have been identified as ILD diagnostic biomarkers.^49^, ^50^ Furthermore, high‐resolution computed tomography (HRCT), and pulmonary function tests are important for RA‐ILD prognosis monitoring. The most common radiological and pathological pattern, UIP, is associated with a poor prognosis and an increased risk of developing acute exacerbations and infections^48^, ^50^; it has also been linked to higher mortality rates.^51^ The prediction model described in patients with SSc‐ILD, which combined the extent of ILD on HRCT and FVC, was equally useful in predicting mortality in patients with RA‐ILD.^52^ According to a systematic review and meta‐analysis, lower DLCO% predicted, lower FVC% predicted, UIP pattern on HRCT, emphysema presence, and acute exacerbation of ILD were associated with an increased risk of mortality in RA‐ILD.^5^ Similarly, the study by Solomon et al.^53^ revealed that the HRCT pattern, a lower FVC% anticipated, and a 10% drop in FVC% predicted from baseline to any point during follow‐up were all independently associated with an increased risk of death. The largest RA‐ILD study in the United Kingdom discovered that baseline gas transfer is a useful tool for detecting ILD, whereas vital capacity (VC) preservation at baseline may indicate limited illness on HRCT. According to the univariate analysis results of a study, anti‐CCP antibody titers were the single most significantly related predictor of RA‐ILD in both sexes. Furthermore, a retrospective analysis revealed that tumor markers CA19‐9, CA125, and CEA, as well as Krebs Von den Lungen‐6 serum levels, were elevated in RA‐ILD and correlated with the severity of ILD, demonstrating their utility as pathogenically important biomarkers.^54^

## 5 CONCLUSION

This is, to the best of our knowledge, the first study to conduct a comprehensive scientometrics analysis of nearly two decades of global RA‐ILD publications. Citations for RA‐ILD research are increasing on an annual basis. We used an information visualization tool to describe RA‐ILD research progress, hotspots, and frontiers over the previous 20 years. These findings can guide RA‐ILD investigation and assist interested researchers in locating potential collaborators. We identified influential authors, institutions, and representative literature in this field. The pathogenesis, treatment, and prediction and prognosis of RA‐ILD are the research directions of RA‐ILD. The similarity research with IPF is direction with respect to the pathogenesis and treatment; antirheumatoid drugs, particularly biological agents and small molecule inhibitors, are prominent therapeutic research directions. Continuous exploration of disease prediction and prognostic factors is critical for improving patient survival rates.

## AUTHOR CONTRIBUTIONS

*Conception and design of the research*: Yue Yang and Xieyu Zhang. *Acquisition of data*: Zixuan Zhang and Xieyu Zhang. *Analysis and interpretation of the data*: Zixuan Zhang, Kai Zhi, and Jiahe Zhao. *Statistical analysis*: Xieyu Zhang, Kai Zhi, Jiahe Zhao. *Obtaining financing*: Wei Cao. *Writing of the manuscript*: Yue Yang, Zixuan Zhang, Xinwen Zhang. *Critical revision of the manuscript for intellectual content*: Wei Cao and Xieyu Zhang. All authors read and approved the final draft.

## CONFLICT OF INTEREST STATEMENT

The authors declare no conflict of interest.

## Supporting information

Supporting information.

## Data Availability

All data generated or analyzed during this study are included in this article. Further enquiries can be directed to the corresponding author.
