# Supplementary material for: Rheumatoid arthritis‐associated interstitial lung disease hotspots and future directions: A Web‐of‐Science based scientometric and visualization study
Source: Immun Inflamm Dis. 2023 Aug 28;11(8):e944. doi: 10.1002/iid3.944 (PMC10461424; doi:10.1002/iid3.944)
Supplement: Supplementary file 1 — Supporting information. [file IID3-11-e944-s001.doc]

**Words that have been merged and deleted**

risk factor, increased risk, scleroderma, progressive systemic sclerosis, sclerosis, multiple sclerosis, connective tissue disorder, mixed connective tissue disease, interstitial pneumonia, usual interstitial pneumonia, nonspecific interstitial pneumonia, pneumonitis, interstitial pneumonitis, organizing pneumonia, hypersensitivity pneumonitis, obliterans organizing pneumonia, bronchiolitis obliterans-organizing pneumonia, bronchiolitis obliterans organizing pneumonia, induced pneumonitis, idiopathic interstitial pneumonia, low dose methotrexate, methotrexate therapy, methotrexate treatment, classification criteria, revised criteria, autoantibody, monoclonal antibody, necrosis factor alpha, tnf alpha, factor alpha, anti tnf therapy, tumor necrosis factor-alpha, tumor necrosis factor-alpha promoter, tnf alpha promoter, tnf alpha blockade, manifestation, feature, clinical presentation, airway inflammation, granulomatous inflammation, extra-articular manifestation, primary sjogrens syndrome, regulatory t cell, cd4(+), cd8(+) t cell, t cell activation, lymphocyte t subset, serious infection, severe infection, intracellulare pulmonary infection, infliximab therapy, mechanism, polymyositis, dermatomyositis, polymyositis dermatomyositis, juvenile dermatomyositis, myositis, inflammatory myositis, childhood dermatomyositis, atopic dermatitis, amyopathic dermatomyositis, bronchoalveolar lavage fluid, prognostic significance, pulmonary hypertension, pulmonary function test, pulmonary function testing, systemic lupus erythematosus, smoking, serum kl 6, etanercept therapy, malignant lymphoma, malignancy, carcinoma, lung cancer, non-small cell lung cancer, non hodgkins lymphoma, resolution computed tomography, high resolution ct, computed tomography, ct finding, high-resolution computed tomography, lung - ct, thin section ct, crohns disease, ulcerative coliti, bowel disease, active crohns disease, colonic mucosa, intestinal inflammation, pulmonary sarcoidosis, wegeners granulomatosis, cutaneous sarcoidosis, granuloma, complicated sarcoidosis, systemic sarcoidosis, progressive pulmonary sarcoidosis, pulmonary nodule, rheumatoid nodule, pulmonary granuloma formation, growth factor beta, transforming growth factor beta 1, transforming growth factor-beta, fibrosing alveolitis, cryptogenic fibrosing alveolitis, ifn gamma, term follow up, cutaneous vasculitis, leukocytoclastic vasculitis, primary vasculitis, churg strauss syndrome, microscopic polyangiitis, necrotizing vasculitis, autoimmune vasculitis, angiitis, cyclosporine, cyclic citrullinated peptide, citrullinated peptide antibody, heart disease, constrictive pericarditis, unstable angina, future myocardial infarction, mycobacterium tuberculosis, c-reactive protein, respiratory bronchiolitis, bronchiolitis obliteran, follicular bronchiolitis, constrictive bronchiolitis, alveolar macrophage, muc5b promoter polymorphism, protein gene, gene polymorphism, promoter variant, gene transfer, 5' flanking region, class ii allele, transcription factor, gene mutation, pleural effusion, parapneumonic effusion, cd95, epithelial cell apoptosis, diffuse alveolar hemorrhage, alveolar hemorrhage, childhood, early childhood, n-3 fatty acid, vcam 1, adhesion molecules icam 1, monitoring liver toxicity, lung - radiography, epstein-barr virus.
